# Delineation of IS*Ecp1* and IS*26*-Mediated Plasmid Fusion Processes by MinION Single-Molecule Long-Read Sequencing

**DOI:** 10.3389/fmicb.2021.796715

**Published:** 2022-02-07

**Authors:** Kaichao Chen, Miaomiao Xie, Edward Wai-Chi Chan, Sheng Chen

**Affiliations:** ^1^Department of Infectious Diseases and Public Health, Jockey Club College of Veterinary Medicine and Life Sciences, City University of Hong Kong, Kowloon, Hong Kong SAR, China; ^2^State Key Laboratory of Chemical Biology and Drug Discovery, Department of Applied Biology and Chemical Technology, The Hong Kong Polytechnic University, Kowloon, Hong Kong SAR, China

**Keywords:** helper plasmid, IS*Ecp1*, IS*15DI*, polymorphism, plasmid fusion

## Abstract

We recently reported the recovery of a novel IncI1 type conjugative helper plasmid which could target mobile genetic elements (MGE) located in non-conjugative plasmid and form a fusion conjugative plasmid to mediate the horizontal transfer of the non-conjugative plasmid. In this study, interactions between the helper plasmid pSa42-91k and two common MGEs, IS*Ecp1* and IS*15DI*, which were cloned into a pBackZero-T vector, were monitored during the conjugation process to depict the molecular mechanisms underlying the plasmid fusion process mediated by insertion sequence (IS) elements. The MinION single-molecule long-read sequencing technology can dynamically reveal the plasmid recombination events and produce valuable information on genetic polymorphism and plasmid heterogeneity in different multidrug resistance (MDR) encoding bacteria. Such data would facilitate the development of new strategies to control evolution and dissemination of MDR plasmids.

## Introduction

Bacterial asexual reproduction is known to involve division of its genetic traits equally into daughter cells to maintain the original phenotypic characteristics. However, bacteria may also evolve by elevating genetic plasticity via horizontal gene transfer (HGT), a process in which beneficial genetic elements are acquired from different species and passed onto the offspring of the recipient strain, whereas harmful genes are prevented from being heritable (Thomas and Nielsen, 2005; Pinilla-Redondo et al., 2018). Three processes of HGT, namely transformation, transduction, and conjugation, have been described to date (Frost et al., 2005). During the transformation process, exogenous genes are passively acquired by the recipient cells and mobile genetic elements (MGE) are not involved. Transduction is often accompanied by bacteriophage invasion, yet this infection event is usually detrimental to the recipient strain (Davison, 1999). In contrast, bacterial conjugation is a much more prevalent HGT mechanism which requires an intricate molecular structure to transfer genetic materials from a donor strain to a recipient strain (Garcillán-Barcia et al., 2009; De La Cruz et al., 2010). Conjugation plays a significant role in dissemination of antimicrobial resistance and virulence-encoding elements in bacteria (San Millan, 2018; Xie et al., 2020). Conjugative plasmids normally harbor genes that encode genetic transfer functions and the Type IV secretion system, which are essential elements that drive the conjugation events. A number of non-conjugative plasmids also encode transfer functions via cooperation with co-resident conjugative plasmids that carry the *oriT* sequence, or via expression of the cognate genes to produce relaxase (Miyano et al., 2018). In previous studies, we reported the discovery of helper plasmids which mediated transmission of non-conjugative plasmids and a chromosomal DNA fragment though fusion mechanism. In these co-integration processes, the insertion sequences (ISs) IS*26* and IS*Pa40* played a critical role in molecular interaction via replicative transposition and homologous recombination, and led to a sharp increase in the incidence of *Salmonella* resistance to ciprofloxacin (Chen et al., 2018, 2019a,b). In addition, these ISs, particularly IS*26*, exhibit a vital function in the spreading of antimicrobial resistance elements among gram-negative strains, as it is normally linked with determinants that confer resistance to various categories of antimicrobial agents (Wrighton and Strike, 1987; Kim and Aoki, 1994), or some class 1 integrons (Miriagou et al., 2005). In this work, we investigated the nature of interactions between helper plasmids and MGEs during the conjugation process of *Salmonella*. Plasmid pSa42-91k is a 91-kbp-conjugative plasmid originated from a strain of *Salmonella* Meleagridis, which exhibited high homology to pSa44-CRO (MH430883) and was considered a helper plasmid. Two prevalent mobile genetic elements, IS*Ecp1* and IS*15DI*, were cloned into the pBackZero-T vector and transferred to a strain known as Sa42, which was then used as the donor strain in conjugation experiments. Recipient strains which have acquired fusion plasmids were subjected to whole genome sequence analysis. Utilizing the long-read sequencing technology, we revealed the functional role of IS*Ecp1* and IS*15DI* in mediating recombination events that enhance the genetic plasticity of the resistance-encoding plasmids during the process of conjugative transfer, and hence the dissemination potential of the resistance genes harbored by the plasmids concerned.

## Materials and Methods

### Cloning of IS*Ecp1* and IS*15DI*

DNA segments were amplified using primer pairs targeting the IS*Ecp1* gene (IS*Ecp1-*F-AGCGTGGTAATGCTGAAAACT and IS*Ecp1-*R-TCCACAGAGCAACACTCAAT) and the IS*15DI* gene (IS*15DI-*F-TGTGGTTAATGCAAAGCGGG and IS*15DI*-R-AAGTCCGCCACATTCGTCTG). The PCR products were ligated to a cloning vector, pBackZero-T, respectively, yielding pBackZero- IS*Ecp1* and pBackZero-IS*15DI*, which were then transformed into *E. coli* DH5α by electroporation. All transformants were selected on LB plates containing 50 μg/mL kanamycin. Transconjugants named DH5α-IS*Ecp1* and DH5α-IS*15DI* were obtained, followed by confirmation of genetic identity through PCR screening with the pair of cloning primers described above.

### Conjugation Experiments

The transmission potential of the IS*Ecp1*, IS*15DI* and *bla*_*CTX–M–*130_ genes was assessed by performing a conjugation experiment using the filter mating method as previously described (Chen et al., 2018). Sa42, Sa42-TC1, and Sa42-TC2 were used as donor strains and the recipient strain was the sodium azide-resistant *E. coli* strain *J53*. The transconjugant Sa42-TC3 was selected on EMB agar containing cefotaxime (2 μg/ml), and sodium azide (100 μg/ml). For donor strains Sa42-TC1 and Sa42-TC2 carrying different pBackZero*-IS* vectors, transconjugants Sa42-TC4 and Sa42-TC5 were selected on EMB agar containing kanamycin (50 μg/ml) and sodium azide (100 μg/ml). Antimicrobial susceptibility of both parental strains and their transconjugants was determined (Table 1).

**TABLE 1:** Phenotypic characteristics of IS*Ecp1* and IS*15DI*–bearing strains and their corresponding transconjugants.

Strain ID	Species	Plasmids	MIC (μg/ml)												
			AMK	CIP	CRO	CTX	KAN	OLA	MRP	NAL	STR	CHL	TET	AMP	CLS
Sa42	*Salmonella*	pSa42-91k	4	0.06	>16	>16	8	16	0.06	8	8	2	2	>64	2
J53	*E. coli*	/	1	0.015	0.03	0.03	1	8	0.03	4	2	0.5	0.5	16	0.5
DH5a	*E. coli*	/	8	0.03	≤0.03	≤0.03	1	1	0.06	32	1	1	0.5	16	0.5
DH5a-T1	*E. coli*	pBackZero-IS*Ecp1*	8	0.03	≤0.03	≤0.03	>128	1	0.06	32	1	1	0.5	64	0.5
DH5a-T2	*E. coli*	pBackZero-IS*15DI*	8	0.03	≤0.03	≤0.03	>128	1	0.06	32	1	1	0.5	64	0.5
Sa42-TC1	*Salmonella*	pSa42-91k, pBackZero-IS*Ecp1*	8	0.03	>16	>16	>128	32	0.12	4	8	1	1	>64	2
Sa42-TC2	*Salmonella*	pSa42-91k, pBackZero-IS*15DI*	8	0.03	>16	>16	>128	32	0.12	4	8	1	1	>64	2
Sa42-TC3	*E. coli J53*	pSa42-91k	1	0.015	>16	>16	1	16	0.12	4	1	4	2	>64	0.5
Sa42-TC4	*E. coli J53*	pSa42-TC4	1	0.015	>16	>16	>128	16	0.12	4	1	4	2	>64	0.5
Sa42-TC5	*E. coli J53*	pSa42-TC5-92k, pSa42-TC5-96k, pSa42-TC5-117k	1	0.015	>16	>16	>128	16	0.12	4	1	4	2	>64	0.5

*AMK, amikacin; CTX, cefotaxime; CIP, ciprofloxacin; KAN, kanamycin; OLA, olaquindox; STR, streptomycin; CRO, ceftriaxone; TET, tetracycline; CHL, chloramphenicol; NAL, nalidixic acid; AMP, ampicillin; MRP, meropenem; CLS, colistin.*

### Pulsed-Field Gel Electrophoresis

Pulsed-field gel electrophoresis (CHEF -MAP -PER System, Bio-Rad Laboratories, Hercules, CA, United States) was performed to confirm the genetic identity of the parental strains and the corresponding transconjugants as described previously (Ribot et al., 2006). All plasmid-bearing transconjugants were analyzed by S1 nuclease PFGE.

### Analysis of Plasmidomes Based on Sequencing Data

Total plasmid samples of strain Sa42 and the corresponding transconjugants were extracted using the Qiagen Midi Plasmid Kit and sequenced with two different platforms, namely the Illumina and Oxford Nanopore Technologies (ONT) MinION platform. Paired-end libraries were constructed and sequenced by using a 300 cycle Illumina NextSeq 500 Kit. Sequencing reads were assembled *de novo* with the SPAdes 3.5 tool (Bankevich et al., 2012). A Rapid Barcoding Sequencing Kit was used to construct the libraries sequenced in a MinION device as previously reported (Li et al., 2018). To determine the structure of the plasmids, Nanopore raw contigs containing pBackZero-T were selected by sequence homology searches with BLAST + makeblastdb (ver. 2.2.28) (Cock et al., 2015) and relevant sequences were extracted from the initial fasta files by faSomeRecords^1^. Long reads assembled from Nanopore were used to align and join contigs acquired from Illumina assembly using the CLC Genomics Workbench v10 (CLC bio, Denmark) and then annotated by the Rapid Annotation using Subsystem Technology (RAST) version 2.0 (Aziz et al., 2008). The EasyFig tool was used to compare and visualize the structures of plasmids and long reads (Alikhan et al., 2011).

## Results and Discussion

### Research Design

**FIGURE 1:**
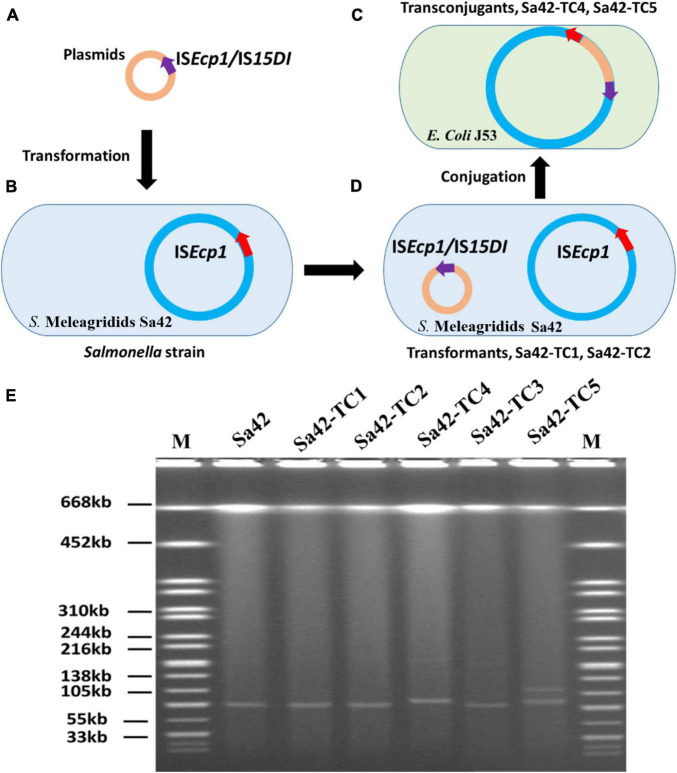
Research design for resolution of the plasmid profile of *Salmonella* strain Sa42 and the corresponding transconjugants by S1-PFGE. **(A)** Plasmids pBackZero-IS*Ecp1* and pBackZero-IS*15DI* were transferred to **(B)** strain Sa42 by transformation and generated **(C)** Sa42-TC1 and Sa42-TC2, respectively, **(D)** which were then conjugated to *E. coli* J53 to produce the transconjugants Sa42-TC4 and Sa42-TC5 that carry fusion plasmids. **(E)** Plasmids in strain Sa42, Sa42-TC1, Sa42-TC2, Sa42-TC3, Sa42-TC4, and Sa42-TC5 by S1-PFGE. Conjugation of Sa42 to *E. coli* J53 produced Sa42-TC3. The sizes of plasmids in Sa42-TC4 and Sa42-TC5 were different from the one in Sa42-TC3. Red arrow depicts original IS*Ecp1* in plasmid pSa42-91k, purple arrow denotes insertion sequences IS*Ecp1* or IS*15DI* being cloned into pBackZero-T vector.

To depict the role of IS*Ecp1* and IS*15DI* (IS*26*) in dissemination of antibiotic resistance genes, these IS elements were first cloned into the pBackZero-T vector to obtain pBackZero-IS*Ecp1* and pBackZero-IS*15DI*. To differentiate the original IS*Ecp1* from the one being cloned into pBackZero-T, only a 341 bp fragment (21–361) of IS*Ecp1* was cloned into pBackZero-T. These two plasmids were then transformed into Sa42, a foodborne *Salmonella* strain, which carried a IncI1 type plasmid, pSa42-91k, that contains the mobile element IS*Ecp1-bla*_*CTX–M–*130_-IS*903.* The pSa42-91k plasmid was able to form fusion plasmid with other antimicrobial resistance (AMR)-encoding plasmids. We therefore used it as a model plasmid to study the fusion process mediated by IS elements. Upon transformation, two transformants were obtained, namely Sa42-TC1 and Sa42-TC2, which carried pSa42-91k/pBackZero-IS*Ecp1* and pSa42-91k/pBackZero-IS*15DI*, respectively. Strains Sa42-TC1 and Sa42-TC2 were then used as donor strains and subjected to conjugation with the recipient *E. coli* strain J53 to obtain transconjugants Sa42-TC4 and Sa42-TC5, which were then subjected to sequencing by both Illumina and Nanopore MinION to assess the conjugation potential of fusion plasmid pSa42-91k/pBackZero-IS*Ecp1* and pSa42-91k/pBackZero-IS*15DI*, as well as to investigate the fusion process (Figure 1).

### *In vitro* Plasmid Fusion and Creation of a Transmission Model

**FIGURE 2:**
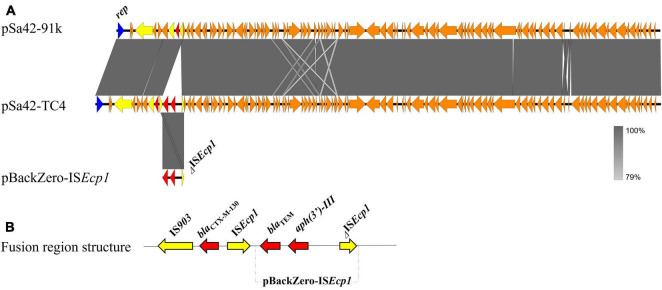
Alignment and structure of the relevant plasmids in the donor strain and transconjugant. **(A)** Linear alignment of plasmids pSa42-91k, pSa42-TC4, and pBackZero-IS*Ecp1* assembled *de novo* by Nanopore long reads. IS elements were highlighted in yellow and drug-resistance genes were depicted in red arrows. **(B)** Structure of pBackZero-I*SEcp1* upon being merged with the plasmid pSa42-91k. Red denoted the resistance genes and yellow denoted the ISs gene.

*Salmonella* Meleagridis Sa42 was resistant to cefotaxime (Table 1). The cefotaxime-resistance phenotype of this strain, which was conferred by *bla*_*CTX–M–*130_, was found to be transferable in a conjugation experiment under the selection of cefotaxime and sodium azide. S1-PFGE revealed that it carried only one plasmid of ∼90 kb, which could be conjugated to *E. coli* J53 under the selection of ceftazidime and sodium azide to produce transconjugant Sa42-TC3, suggesting that it was a conjugative plasmid (Figure 1E). This plasmid was subjected to whole plasmid sequencing using both the Illumina and Nanopore sequencing platform to obtain the complete plasmid map. Our data showed that this plasmid was 91,229 bp in length, exhibited a GC content of 50.9%, and comprised 98 putative open reading frames (ORFs), most of which were responsible for conjugation, replication, partition and other plasmid maintenance functions. A 530 bp β-lactamase gene *bla*_*CTX–M–*130_ franked by *ISEcp1* was also found in plasmid pSa42-91k at position 7434-8039 (Figure 2A).

We next cloned IS*Ecp1* and IS*15DI* into the pBackZero-T vector to produce pBackZero-IS*Ecp1* and pBackZero-IS*15DI*, respectively, followed by conjugation to the pSa42-91k-bearing strain *S.* Meleagridis Sa42 to produce Sa42-TC1 and Sa42-TC2. The conjugation experiment was further performed to obtain Sa42-TC4 and Sa42-TC5, which were *E. coli* J53 which have acquired a variety of fusion plasmids formed by interaction between pSa42-91k and pBackZero-IS*Ecp1* or pBackZero-IS*15DI*. It was expected that Sa42-TC4 and Sa42-TC5 contained the fusion plasmid of pSa42-91k and pBackZero-IS*Ecp1* or pBackZero-IS*15DI*. S1-PFGE was performed on these five transconjugants, with results showing that Sa42, Sa42-TC1, and Sa42-TC2 carried only one plasmid with a size similar to that of pSa42-91k. However, Sa42-TC4 carried only one plasmid but the size was slightly larger than that of plasmid pSa42-91k, suggesting that this plasmid should be the fusion product of pSa42-91k and pBackZero-IS*Ecp1*. Interestingly, Sa42-TC5 carried two plasmids of different sizes and both were larger than pSa42-91k. These two plasmids should be the fusion product of pSa42-91k and pBackZero-IS*15DI*, but exhibited different structural arrangement (Figure 1E). Antimicrobial susceptibilities were determined for Sa42, Sa42-TC1, Sa42-TC2, Sa42-TC3, Sa42-TC4, Sa42-TC5 to confirm that these transconjugants exhibited the right antimicrobial resistance phenotypes encoded by the plasmids they harbored, namely ceftazidime resistance for pSa42-91k and kanamycin resistance for pBackZero-IS*Ecp1* and pBackZero-IS*15DI*. Antimicrobial susceptibility results showed that the resistance phenotypes of these transconjugants matched well with those encoded by the plasmids that they harbored (Table 1).

### Genetic Features of Conjugative Fusion Plasmids in Sa42-TC4 and Sa42-TC5

The plasmids harbored by *E. coli* DH5α-T1 and DH5α-T2 were sequenced by Illumina, with results showing that pBackZero-IS*Ecp1* from DH5α-T1 was 3,596 bp in length and contained the pBackZero-T vector and a 341 bp DNA fragment containing IS*Ecp1*; pBackZero-IS*15DI* from DH5α-T2 was 4,157 bp in length, containing the pBackZero-T vector and a 820 bp DNA fragment harboring IS*15DI*. Both plasmids had a GC content of 47.8%, comprised 3 coding sequences (CDSs) and harbored the kanamycin resistance gene.

**FIGURE 3:**
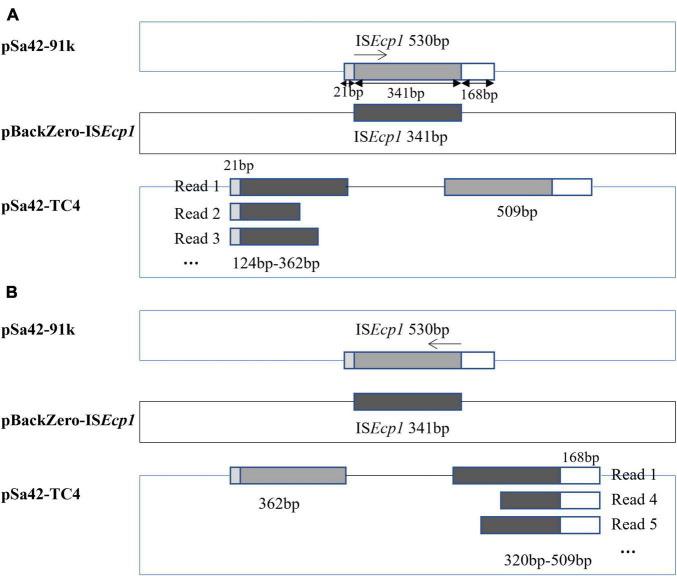
Mechanism of IS*Ecp1*–mediated homologous recombination depicted by analysis of Nanopore long-reads. IS*Ecp1* was found to exhibit a size of 530 bp in pSa42-91k. A fragment of IS*Ecp1* was cloned into pBackZero-T vector (depicted in black). Such fragment exhibited sequence homology with IS*Ecp1* from pSa42-91k (depicted in gray). A 21 bp and 168 bp region were located at each terminal. **(A)** When the homologous recombination process started from the left, various sizes of IS*Ecp1* linked with the 21 bp sequence were observed in Nanopore long reads; oppositely oriented terminal side of a 509 bp constant size could be detected in another IS*Ecp1*. **(B)** Homologous recombination began in the opposite direction, Nanopore long reads were also found to contain various sizes of I*SEcp1* at 341 bp, which were connected to a 168 bp fragment in the original IS*Ecp1*, whereas another part of 362 bp in the original IS*Ecp1* whose size was stable and not variable was located in the other end.

In a previous study, IS*Ecp1* was found to mediate genetic transposition events that involve homologous recombination (Zong et al., 2010). Using the Illumina and Nanopore sequencing platforms, complete sequence of the plasmid in Sa42-TC4 was obtained. The plasmid in Sa42-TC4, designated as pSa42-TC4, was a fusion plasmid of pBackZero-IS*Ecp1* and pSa42-91k (Figure 2). The fusion site was found to be at IS*Ecp1*, in which a structure of IS*Ecp1*-pBackZero-IS*Ecp1* was replaced by the original IS*Ecp1* in plasmid pSa42-91k. To differentiate between the IS*Ecp1* element harbored by plasmid pSa42-91k from the one we cloned to pBackZero-T, the latter was designed to be shorter than the one in pSa42-91k, which included 21–362 bp of the full length of IS*Ecp1* (Figure 3A). This design will lead to production of IS*Ecp1* with a size of 341 bp in pBackZero-T and 530 bp in pSa42-91k, respectively (Figure 3A).

Unlike IS*Ecp1*, IS*15DI* was found to belong to the IS*6* family and exhibited genetic characteristics similar to those of IS*26*, which can undergo replicative transposition during the co-integration process. In this study, two different sizes of plasmids from strain Sa42-TC5 were observed by S1-PFGE, indicating that IS*15DI* may target different insertion sites during conjugation. Nanopore long reads sequencing was performed to solve this problem, with sequencing reads being assembled *de novo* by Canu (Koren et al., 2017). Complete sequencing of the large plasmid was obtained, for which a size of 117,221 bp was consistent with the S1-PFGE result. The plasmid contained 147 CDSs with a GC content of 50.8% and was designated as plasmid pSa42-TC5-117k. Compared with the original plasmid pSa42-91k, an IS*15DI* element and an IS*15DI-*pBackZero-IS*15DI* element were found to be inserted into the *traJ* and *traF* genes in plasmid pSa42-TC5-117k, respectively. Another fusion plasmid in strain Sa42-TC5, designated as pSa42-TC5-96k, was formed by insertion of the IS*15DI-*pBackZero-IS*15DI* into the *traF* gene of pSa42-91k, which was 95,624 bp in length, contained 126 CDSs and exhibited a GC content of 50.8%. However, an extra ∼92 kb plasmid could be identified from our sequencing data and was designated pSa42-TC5-92k. This plasmid could not be observed in S1-PFGE as its size was similar to that of plasmid pSa42-TC5-96k. Plasmid pSa42-TC5-92k was 91,784 bp in length, contained only one IS*15DI* inserted into the *traJ* gene, and exhibited a GC content of 50.9%. For the fusion plasmid pSa42-TC5-117k, in addition to an insertion event in the *traJ* gene, another insertion site was also observed in *traF* in which a nearly 20 kb DNA sequence upstream of *traJ* was found to be repeatedly inserted beyond the *traJ* gene, which was responsible for causing an increase in the plasmid size to around ∼117 kb.

### Mechanism That Gives Rise to IS*Ecp1* Polymorphisms and Enhances Dissemination Potential

To depict the detailed mechanism of interaction between the IS*Ecp1* elements located in two plasmids, MinION nanopore long reads were generated from Sa42-TC4 and reads baring the fusion region were extracted for further analysis (Table 2). Nanopore raw fasta reads (>50 kb) were selected and the chromosomal contamination reads were removed by matching against the pSa42-91k reference plasmid via BLASTN. To check the fusion structure in each read, two genes harbored by the plasmid pBackZero-T including *bla*_*TEM*_ and *aph(3′)-III* were used as the marker genes to filter out reads that did not span the fusion regions of IS*Ecp1-*pBackZero-IS*Ecp1.* Among the Nanopore reads from sample Sa42-TC4, 256 out of 376 reads were found to harbor complete structure of IS*Ecp1-*pBackZero-IS*Ecp1* in various sizes. Different sizes of reads were selected for BLASTN analysis against relevant sequences in donor strains (Figure 3). IS*Ecp*1 in IS*Ecp1*-pBackZero-IS*Ecp1* structures of various sizes was observed among different reads and shown to be polymorphic by Rast tools (Overbeek et al., 2013). These reads could be grouped into two categories based on alignment to two different sizes of IS*Ecp1*, which originated from pBackZero-T and pSa42-91k, respectively. One category of reads contained a 509 bp IS*Ecp1*, with the first 21 bp of DNA located on the right of the reads, whereas various sizes of IS*Ecp1* sequences ranging from 124 to 362 bp were found located at the left side of the reads (Figure 3A). IS*Ecp1* elements located at the left are often truncated at different sites. These data suggested that (1) fusion of pBackZero-IS*Ecp1* to pSA42-91k was mediated by homologous recombination; (2) the truncated IS*Ecp1* located in pBackZero aligned to the full length of IS*Ecp1* at 21 bp site, which initiated the homologous recombination; and (3) the length of the homologous region varied from 103 bp to the full length of the truncated IS*Ecp1* (341 bp); and upon insertion of pBackZero-IS*Ecp1* into the fusion plasmid, the IS*Ecp1* was duplicated (Figure 3B).

**TABLE 2:** Statistical summary of MinION Nanopore long reads generated from plasmid samples of strains Sa42-TC4 and Sa42-TC5.

Sequence statistic	pSa42-TC4 reads	pSa42-TC4 reads (>50 kb)	pSa42-TC5 reads	pSa42-TC5 reads (>50 kb)
No. of sequences	15,483	376	19,488	433
Sequence length (bp)	112,500,802	29,167,802	132,510,505	33,297,717
Sequence N base (bp)	0	0	0	0
Average length (bp)	17,487	77,573.94	6,799.59	76,900.04
N50 length (bp)	12,140	97,133	14,750	88,039
N90 length (bp)	7,985	80,484	3,532	55,673
Maximum length (bp)	159,040	159,040	150,465	150,465
Minimum length (bp)	500	50,140	500	50,048
(G + C)/(G + C + A + T) (%)	49.88	49.85	49.93	50.11

*Reads (>50 kb) were extracted for further analysis of ISEcp1 activities and IS15DI target sites.*

Another category of reads had a stable 362 bp region of IS*Ecp1* located on the left, whereas the right side of IS*Ecp1* exhibited polymorphism, with sizes ranging from 320∼509 bp. Sequence analysis showed that the truncated region on the left covered base 1∼362 of IS*Ecp1*, yet the terminal region of the truncated IS*Ecp1* was located in pBackZero-IS*Ecp1*. The right hand side of IS*Ecp1* existed in various sizes and always ended at position 530, even though the start sites in IS*Ecp1* were variable. These findings suggest that the 3′-terminal of the truncated IS*Ecp1* could also align with the IS*Ecp1* element located in a site at 362 bp in plasmid pSa42-91k, as well as the full length of IS*Ecp1.* Homologous recombination could occur at short sequence at any site within a 152 bp fragment (from position 210 to 362) and regions up to the start codon of IS*Ecp1.* Upon homologous recombination, the rest of IS*Ecp1* (from 1 to 362) will remain at the left side of the fusion plasmid (Figure 3B). Among the reads from nanopore, the most dominant reads were 362 bp of the truncated IS*Ecp1* at the left side and 590 bp at the right side, which could be generated through molecular interaction by targeting either the 5′- or 3′-terminal of the truncated IS*Ecp1* located in pBackZero-IS*Ecp1* or the IS*Ecp1* located in pSa42-91k. Based on our understanding of the homologous recombination process, we believe that both copies of IS*Ecp1*, located in pBackZero-IS*Ecp1*, and pSA42-91k were identical. In this case, upon homologous recombination, the IS*Ecp1* located at the left could become variable in size, whereas the IS*Ecp1* element located at the right remains full length.

### Mechanism of Generating IS*15DI* Polymorphisms and Enhancing Dissemination Potential

**FIGURE 4:**
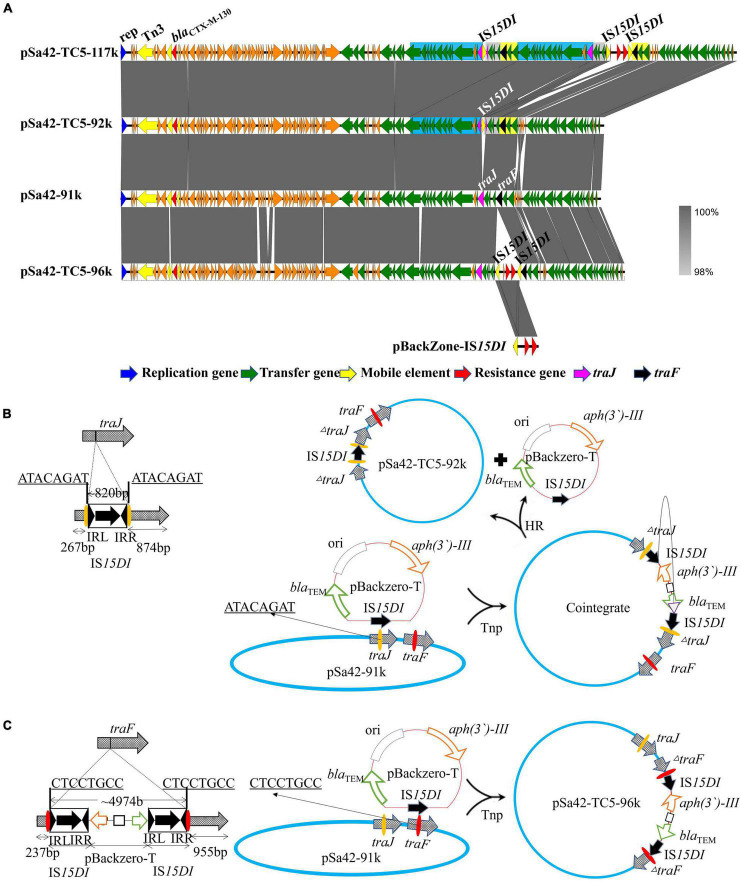
Alignment of three plasmids in transconjugant Sa42-TC5 and schematic representation of the two insertion events. **(A)** Alignment of various plasmids harbored by strain Sa42-TC5. Plasmids pSa42-TC5-117k, pSa42-TC5-92k, and pSa42-TC5-96k were obtained from transconjugant Sa42-TC5; pSa42-TC5-117k had two target site duplications (TSD) and was assembled *de novo* with Canu, the slight blue, gray, and yellow color depicted various repeated regions. Plasmids pSa42-TC5-92k and pSa42-TC5-96k with one TSD were selected directly from nanopore long reads and calibrated by matching with known plasmid sequences. **(B)** Genetic mechanisms of insertion of IS*15DI* into *traJ*. IS*15DI* led to an attack on the hot spot in the *traJ* gene. Resolution of *traJ* via transposase (Tnp) mediated cointegration, which was followed by homologous recombination (HR). **(C)** Proposed IS element-mediated fusion in *traF* only via replicative transposition event at the hot spot (CTCCGTCC). Target site duplications from different genes were underlined with bold letters. The left and right inverted repeats 14 bp (IRL and IRR) of IS*15DI* were shown in black triangles. IS*15DI* was denoted in black with an arrow indicating the orientation and length of the transposase gene.

Similarly, nanopore long reads containing IS*15DI* were extracted and analyzed by the aforementioned method (Table 2). Nanopore raw fasta reads (>50 kb) were also selected and chromosomal contamination reads were screened out by mapping against the pSa42-91k reference plasmid via BLASTN. Unlike IS*Ecp1*, IS*15DI* was known to undergo replicative transposition during the co-integration process like IS*26*, but IS*15DI* did not exist in the original plasmid pSa42-91k, so it can be regarded as a marker for direct selection from raw reads. Among the nanopore reads from Sa42-TC5, 222 out of 433 reads were found to harbor complete IS*15DI* sequences. Different forms of reads of various sizes were observed in the case of IS*Ecp1*, among which three type of reads could be identified. The first type was a structure in which IS*15DI*-pBackZero-IS*15DI* was inserted into the *traF* gene by replicative transposition (Figure 4C; Arthur and Sherratt, 1979). During the process, the IS*15DI* element cleaved both terminal inverted repeats (TIRs), resulting in the formation of nicks in both strands of DNA and generating 3-Diols groups (3 = -OH) that attack the hot spot of the *traF* gene, leading to formation of a Shapiro intermediate. The DNA complex then underwent intermolecular replicative transposition, in which the target site was duplicated. The second type involved insertion of one copy of IS*15DI* into the *traJ* gene. The process probably involved the insertion of IS*15DI-*pBackZero-IS*15DI* into the *traJ* gene and then the element was excised out, leaving a single scar copy of IS*15DI* in the *traJ* gene (Figure 4B). Interestingly, a 8 bp target site duplication sequence (ATACAGAT) was found to flank the IS*15DI* elements, indicating that this process may occur in two steps. The first step was mediated by the attack at the hot spot of the *traJ* gene by the insertion element IS*15DI*, leading to insection of IS*15DI*-pBackZero-IS*15DI* into the *traJ* gene, followed by the excision of pBackZero-IS*15DI* from the fusion plasmid through homologous recombination, leaving one single copy of the IS*15DI* scar in *traJ* gene (Harmer et al., 2020). This type of plasmid contained only one copy IS*15DI*, which existed in a circular form observable in Nanopore long reads, but the number of such plasmid was too low to be seen in S1-PFGE. The third type is more complicated. Insertion events occurred in both the *traF* and *traJ* genes and duplication of a DNA fragment from the pSa42-91k led to the formation of a plasmid of a much larger size (∼117 kb) designated as pSa42-TC5-117k (Figure 4A). The mechanism of DNA duplication in this type of plasmid is not clear and entails further investigation.

## Conclusion

Insertion of MDR-encoding mobile elements into the chromosome and plasmids has been widely reported following the wide usage of the whole genome sequencing (WGS) technology. With the increasing use of MinION nanopore sequencing technology and Illumina sequencing, IS element-mediated plasmid fusion events have also been increasingly reported. However, most of these previous studies only reported the final plasmid sequences based on the assembly of the most dominant reads and contigs generated from the sequencing platforms. The underlying mechanisms of the fusion and integration processes remain unknown. In this study, we have designed a set of experiments to investigate the molecular mechanisms underlying two of the most common fusion processes mediated by IS*Ecp1* and IS*26*-like elements, which involve homologous recombination and replicative transposition, respectively. Our data showed that both the 5′- or 3′-terminal of IS*Ecp1* could attack another copy of IS*Ecp1* through interaction with homologous sequences as short as 100 bp, resulting in the formation of a truncated IS*Ecp1*. This finding helps explain why IS elements are commonly found in MDR-encoding genetic fragments. The IS*15DI* element mainly attacks the hot spots located in different target genes and mediates the conjugation process of plasmids that contain the *traF* and *traJ* genes. The hot spot site seems to be conservative for specific IS element, whereas the type of the hot spot site determines the mode of the integration. Findings in this study showed that detailed analysis of Nanopore reads would enable us to depict the dynamic DNA fusion processes mediated by different IS. This approach can be applied to study the mechanism of DNA fusion process mediated by other important IS elements.

## Data Availability Statement

The datasets presented in this study can be found in online repositories. The names of the repository/repositories and accession number(s) can be found below: https://www.ncbi.nlm.nih.gov/genbank/, MW567497, https://www.ncbi.nlm.nih.gov/genbank/, MW567498, https://www.ncbi.nlm.nih.gov/genbank/, MW881232, https://www.ncbi.nlm.nih.gov/genbank/, MW881230, and https://www.ncbi.nlm.nih.gov/genbank/, MW881231.

## Author Contributions

KC performed sequencing and bioinformatic analysis. MX helped with conjugation and other experiments. EC, KC, and SC participated in research design and manuscript writing. SC supervised the project. All authors contributed to the article and approved the submitted version.

## Conflict of Interest

The authors declare that the research was conducted in the absence of any commercial or financial relationships that could be construed as a potential conflict of interest.

## Publisher’s Note

All claims expressed in this article are solely those of the authors and do not necessarily represent those of their affiliated organizations, or those of the publisher, the editors and the reviewers. Any product that may be evaluated in this article, or claim that may be made by its manufacturer, is not guaranteed or endorsed by the publisher.

## Footnotes

1
https://github.com/santiagosnchez/faSomeRecords

## References

[B1] Alikhan N.-F. Petty N. K. Zakour N. L. B. Beatson S. A. (2011). BLAST Ring Image Generator (BRIG): simple prokaryote genome comparisons. *BMC Genomics* 12:1. 10.1186/1471-2164-12-402 21824423 PMC3163573

[B2] Arthur A. Sherratt D. (1979). Dissection of the transposition process: a transposon-encoded site-specific recombination system. *Mole. Gen. Genet. MGG* 175 267–274. 10.1007/BF00397226 392228

[B3] Aziz R. K. Bartels D. Best A. A. DeJongh M. Disz T. Edwards R. A. (2008). The RAST Server: rapid annotations using subsystems technology. *BMC Genomics* 9 1–15. 10.1186/1471-2164-9-75 18261238 PMC2265698

[B4] Bankevich A. Nurk S. Antipov D. Gurevich A. A. Dvorkin M. Kulikov A. S. (2012). SPAdes: a new genome assembly algorithm and its applications to single-cell sequencing. *J. Comp. Biol.* 19 455–477. 10.1089/cmb.2012.0021 22506599 PMC3342519

[B5] Chen K. Dong N. Chan E. W.-C. Chen S. (2019a). Transmission of ciprofloxacin resistance in *Salmonella* mediated by a novel type of conjugative helper plasmids. *Emerg. Microb. Infect.* 8 857–865. 10.1080/22221751.2019.1626197 31169081 PMC6566993

[B6] Chen K. Dong N. Zhao S. Liu L. Li R. Xie M. (2018). Identification and characterization of conjugative plasmids that encode ciprofloxacin resistance in *Salmonella*. *Antimicrob. Agents Chemother.* 62:8. 10.1128/AAC.00575-18 29760137 PMC6105805

[B7] Chen K. Wai Chi, Chan E. Chen S. (2019b). Evolution and transmission of a conjugative plasmid encoding both ciprofloxacin and ceftriaxone resistance in *Salmonella*. *Emerg. Microb. Infect.* 8 396–403. 10.1080/22221751.2019.1585965 30896347 PMC6455229

[B8] Cock P. J. Chilton J. M. Grüning B. Johnson J. E. Soranzo N. (2015). NCBI BLAST+ integrated into Galaxy. *Gigascience* 4:3747. 10.1186/s13742-015-0080-7 26336600 PMC4557756

[B9] Davison J. (1999). Genetic exchange between bacteria in the environment. *Plasmid* 42 73–91. 10.1006/plas.1999.1421 10489325

[B10] De La Cruz F. Frost L. S. Meyer R. J. Zechner E. L. (2010). Conjugative DNA metabolism in Gram-negative bacteria. *FEMS Microbiol. Rev.* 34 18–40. 10.1111/j.1574-6976.2009.00195.x 19919603

[B11] Frost L. S. Leplae R. Summers A. O. Toussaint A. (2005). Mobile genetic elements: the agents of open source evolution. *Nat. Rev. Microbiol.* 3 722–732. 10.1038/nrmicro1235 16138100

[B12] Garcillán-Barcia M. P. Francia M. V. De La Cruz F. (2009). The diversity of conjugative relaxases and its application in plasmid classification. *FEMS Microb. Rev.* 33 657–687. 10.1111/j.1574-6976.2009.00168.x 19396961

[B13] Harmer C. J. Pong C. H. Hall R. M. (2020). Structures bounded by directly-oriented members of the IS26 family are pseudo-compound transposons. *Plasmid* 2020:102530. 10.1016/j.plasmid.2020.102530 32871211

[B14] Kim E.-H. Aoki T. (1994). The transposon-like structure of IS26-tetracycline, and kanamycin resistance determinant derived from transferable R plasmid of fish pathogen, Pasteurella piscicida. *Microb. Immunol.* 38 31–38. 10.1111/j.1348-0421.1994.tb01741.x 8052160

[B15] Koren S. Walenz B. P. Berlin K. Miller J. R. Bergman N. H. Phillippy A. M. (2017). Canu: scalable and accurate long-read assembly via adaptive k-mer weighting and repeat separation. *Genome Res.* 2017:215116. 10.1101/gr.215087.116 28298431 PMC5411767

[B16] Li R. Xie M. Dong N. Lin D. Yang X. Wong M. H. Y. (2018). Efficient generation of complete sequences of MDR-encoding plasmids by rapid assembly of MinION barcoding sequencing data. *GigaScience* 7:gix132. 10.1093/gigascience/gix132 PMC5848804 29325009

[B17] Miriagou V. Carattoli A. Tzelepi E. Villa L. Tzouvelekis L. S. (2005). IS26-associated In4-type integrons forming multiresistance loci in enterobacterial plasmids. *Antimicrob. Agents Chemother.* 49 3541–3543. 10.1128/AAC.49.8.3541-3543.2005 16048979 PMC1196216

[B18] Miyano M. Tanaka K. Ishikawa S. Takenaka S. Miguel-Arribas A. Meijer W. J. (2018). Rapid conjugative mobilization of a 100 kb segment of Bacillus subtilis chromosomal DNA is mediated by a helper plasmid with no ability for self-transfer. *Microb. Cell Fact.* 17 1–10. 10.1186/s12934-017-0855-x 29374463 PMC5787278

[B19] Overbeek R. Olson R. Pusch G. D. Olsen G. J. Davis J. J. Disz T. (2013). The SEED and the Rapid Annotation of microbial genomes using Subsystems Technology (RAST). *Nucleic Acids Res.* 42 D206–D214. 10.1093/nar/gkt1226 24293654 PMC3965101

[B20] Pinilla-Redondo R. Cyriaque V. Jacquiod S. Sørensen S. J. Riber L. (2018). Monitoring plasmid-mediated horizontal gene transfer in microbiomes: recent advances and future perspectives. *Plasmid* 99 56–67. 10.1016/j.plasmid.2018.08.002 30086339

[B21] Ribot E. M. Fair M. Gautom R. Cameron D. Hunter S. Swaminathan B. (2006). Standardization of pulsed-field gel electrophoresis protocols for the subtyping of *Escherichia coli* O157: H7, *Salmonella*, and *Shigella* for PulseNet. *Foodbourne Pathog. Dis.* 3 59–67. 10.1089/fpd.2006.3.59 16602980

[B22] San Millan A. (2018). Evolution of plasmid-mediated antibiotic resistance in the clinical context. *Trends Microb.* 26 978–985. 10.1016/j.tim.2018.06.007 30049587

[B23] Thomas C. M. Nielsen K. M. (2005). Mechanisms of, and barriers to, horizontal gene transfer between bacteria. *Nat. Rev. Microb.* 3 711–721. 10.1038/nrmicro1234 16138099

[B24] Wrighton C. J. Strike P. (1987). A pathway for the evolution of the plasmid NTP16 involving the novel kanamycin resistance transposon Tn4352. *Plasmid* 17 37–45. 10.1016/0147-619x(87)90006-0 3033719

[B25] Xie M. Chen K. Ye L. Yang X. Xu Q. Yang C. (2020). Conjugation of virulence plasmid in Clinical *Klebsiella pneumoniae* strains through formation of a fusion plasmid. *Adv. Biosyst.* 4:1900239. 10.1002/adbi.201900239 32293159

[B26] Zong Z. Partridge S. R. Iredell J. R. (2010). ISEcp1-mediated transposition and homologous recombination can explain the context of blaCTX-M-62 linked to qnrB2. *Antimicrob. Agents Chemother.* 54 3039–3042. 10.1128/AAC.00041-10 20421399 PMC2897309

